# Impact of Type of Sugar Beet Pectin–Sodium Caseinate Interaction on Emulsion Properties at pH 4.5 and pH 7

**DOI:** 10.3390/foods10030631

**Published:** 2021-03-17

**Authors:** Zhang Juyang, Bettina Wolf

**Affiliations:** Division of Food Sciences, School of Biosciences, University of Nottingham, Sutton Bonington Campus, Loughborough LE12 5RD, UK; b.wolf@bham.ac.uk

**Keywords:** emulsion, sugar beet pectin, sodium caseinate, protein–polysaccharide conjugates, interfacial tension, shear oscillatory rheology

## Abstract

Equal parts of sugar beet pectin and sodium caseinate were interacted through electrostatic attraction, enzymatic crosslinking, and the Maillard reaction to prepare three oil-in-water emulsifier systems. Oil-in-water emulsions (10%) were processed via high shear overhead mixing at the natural pH of the emulsifier systems, followed by pH adjustment to pH 4.5 and pH 7. The emulsions were stable against coalescence, except for a slight increase in the mean droplet size for the enzymatic cross-liked emulsion at pH 4.5 over a 14-day storage period. This emulsion also showed the lowest absolute zeta (ζ)-potential value of near 30 mV. The Maillard interaction emulsifier system resulted in larger droplet sizes compared to the other two emulsifier systems. Small deformation oscillatory shear rheology assessment of the emulsion cream phases revealed an impact of the emulsifier system design at pH 4.5.

## 1. Introduction

Proteins, one of the most common naturally occurring high-molecular-weight emulsifiers, are widely used as food-emulsifying and -stabilization ingredients. Casein is such an ingredient and, in addition to being naturally present in dairy products such as cheese and yoghurt, it can be found in products such as non-dairy creams, coffee whiteners, soups, puddings, custards, and sausages. Casein has a strong affinity to hydrophobic surfaces [1] and is capable of emulsion stabilization at neutral pH [2]. At acidic pH close to the protein’s isoelectric point (pH ≈ 4.6), casein-stabilized emulsions are unstable due to the suppression of electrostatic repulsion and the collapse of the steric layer of the casein-coated droplets [3,4]. These challenges can be overcome by conjugating or complexing casein with a pectin [5]. Sugar beet pectin (SBP), despite being a weak gelling agent, possesses better emulsifying properties than other pectins [6], and was therefore selected in this study. The proteinaceous moiety in SBP adsorbs into the surface of oil-in-water emulsion droplets as an anchor, decreasing the interfacial tension between the water and oil phases [7]. SBP is also interesting as a formulation ingredient as it possesses antioxidant and bioactive properties through the presence of ferulic acid (FA) [8,9]. FA is a phenolic compound of the formula of C_10_H_10_O_4_ [10]. This particular phenolic acid is linked to polysaccharides via esterification and is a necessary structural component of the cell wall [8,11].

Protein–polysaccharide conjugates are already applied in food products [12,13], not only as emulsifier [14], but also encapsulation agent [15,16], or fat replacer [17]. Additional drivers for choosing protein–polysaccharide conjugates over other food ingredients with similar functionality may include also of low allergenicity, and biodegradability [13]. As charged high-molecular-weight materials, protein–polysaccharide conjugates provide emulsion stability through steric, and electrostatic stabilization of the emulsion interface [3,18]. They may occur naturally, or be created through charge-induced physical interaction between the protein and the polysaccharide, heat treatment, creating Maillard conjugates, or enzymatic cross–linking [12,19]. Many studies exist understanding the stability of emulsions stabilized by protein–polysaccharide conjugates [5,20,21,22,23,24], but to the best of our knowledge no comparative study between the three types of interaction methods applied to the same system has been published to-date. 

Here, all three aforementioned types of fabricated SBP–sodium caseinate (SC) emulsifier systems were considered, namely electrostatically stabilized (P), enzyme (laccase)-catalyzed crosslinked (E), and Maillard reaction (M) complexes. Previously, we introduced these emulsifier systems and reported their physico-chemical properties [25]. The three emulsifier systems exhibited different aggregation properties when in dispersion at pH 4.5 and pH 7. Acidic and neutral pH are both relevant for food emulsions. The E complex was the most acid-tolerant, while the M complex was the least acid-tolerant, when compared at pH 4.5. All three systems showed similar aggregation properties at pH 7. This follow-up study was dedicated to understanding the functional properties of these conjugates, in view to food industry applications. Therefore, we investigated their interfacial tension, their capability of stabilizing emulsions, and the physico-chemical properties of the emulsions including droplet size, zeta (ζ)-potential, and rheological properties. Due to the instability of the M complexes at pH 4.5, we limited the interfacial tension analysis to pH 7. In terms of the rheological properties, we confined our study to the viscoelastic properties of the emulsion cream phase to gain maximum information about the impact of the interfacial design of the emulsions on their properties.

## 2. Materials and Methods

### 2.1. Materials and Stock Solutions

The main materials used to prepare the emulsifiers were SBP donated by Herbstreith & Fox KG (Neuenbürg, Germany) and SC (Acros, NJ, USA). The citric acid monohydrate and sodium citrate dihydrate used for the preparation of citrate buffers (pH 5), the hydrochloric acid and sodium hydroxide to adjust the pH using 1 M solutions of each, and the sodium azide to prevent microbial spoilage of the samples were obtained from Thermo Fisher Scientific (Loughborough, UK). Sunflower oil was purchased from a local supermarket. Florisil (<200 mesh, fine powder) to remove surface-active molecules in the sunflower oil and laccase enzyme was purchased from Sigma-Aldrich (St. Louis, MI, USA). The laccase activity was reported as 0.87 units per milligram (AU) of the enzyme. Deionized water (electrical conductivity <2 µS·cm^−1^), produced on-site, was used throughout.

Stock solutions of SBP and SC were prepared as follows. Initially, a 50 mM citrate buffer at pH 5 was prepared by diluting 0.1 M citrate buffer (pH 5) with the appropriate amount of water. The 0.1 M citrate buffer was prepared by mixing 20.5 mL of 0.1 M citric acid and 29.5 mL of 0.1 M sodium citrate with 50 mL of water on a magnetic stirrer at 500 rpm and 25 °C for 30 min. Then 1% *w/w* SC and 1% *w/w* SBP were added to water and mixed overnight while stirring at 500 rpm and 25 °C to ensure full hydration. Finally, 1:1 SBP:SC stock solutions with a total polymer content of 0.4% *w/w* were prepared by mixing the appropriate amounts of stock dispersions with water, 10 g of citrate buffer (pH 5), and 0.02% *w/w* sodium azide.

### 2.2. Preparation of the Emulsifier Systems

**Figure 1:**
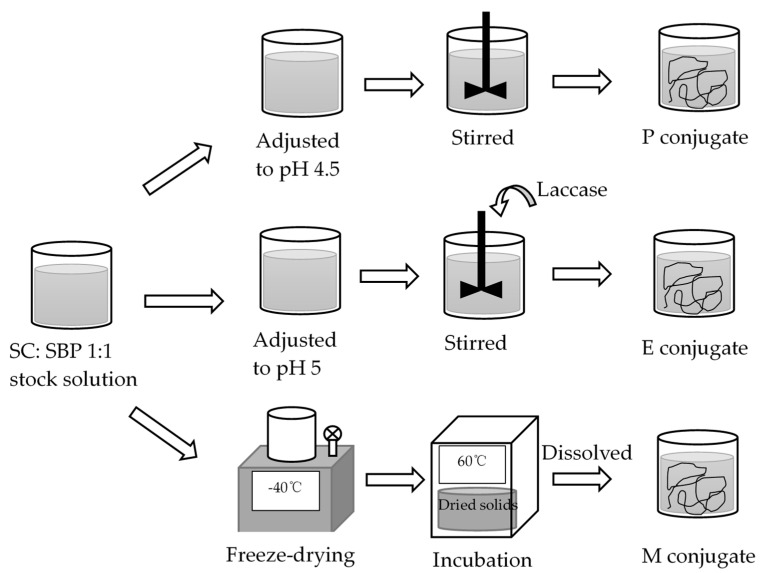
Flow diagram of the preparation of the electrostatically stabilized (P), enzyme (laccase)-catalyzed crosslinked (E), and Maillard reaction (M) conjugates. SBP, sugar beet pectin; SC, sodium caseinate.

The preparation method for each of the three SBP–SC emulsifier systems was exactly the same as in our previous study [25], and is summarized in (Figure 1). Electrostatically stabilized SBP–SC complexes (P complexes) were prepared by adjusting the pH of the SBP–SC stock solution to 4.5 through the addition of either 1 M HCl or 1 M NaOH, as appropriate. This was stirred at 500 rpm and 25 °C for at least 6 h to ensure complete formation of the SBP–SC complexes, confirmed visually through the mixture changing color to white. To prepare the laccase-catalyzed SBP–SC complexes (E complexes), the SBP–SC dispersion was prepared using 10 g of citrate buffer (50 mM, pH 5) containing 5 AU laccase enzyme. After this, the SBP–SC mixture was placed in a magnetic stirrer at 500 rpm and 25 °C for 2 h to ensure a complete enzymatic reaction. After enzymatic catalysis, the dispersion color changed from milky white to light brown. Finally, the Maillard reaction complexes (M complexes) were prepared as follows. SBP and SC were initially dissolved individually in deionized water (solid:liquid ratio of 1:25), then mixed at a ratio of 1:1 by weight while stirring at 700 rpm and 25 °C for 2 h, followed by freeze-drying. The mixture was pre-frozen at −80 °C before transferring into a freeze dryer (Edwards, Super Modulyo, UK) set to operate at −40 °C and 2–7 10^−2^ bar. The dried solids were placed in a desiccator containing a saturated KBr dispersion and incubated for 48 h in a cabinet (Sanyo/Gallenkamp cabinet, model CF4) at 60 °C and 79% relative humidity. The complexes were stored in disposable polypropylene containers at 2 °C until further use. The M emulsifier dispersions were prepared by dissolving 0.4 g of the freeze-dried solids in the appropriate amount of deionized water, containing 0.02% *w/w* sodium azide and 10 g of citrate buffer (50 mM, pH 5). The dispersion was then adjusted to pH 7 by the addition of the appropriate amount of 1 M NaOH, placed on a magnetic stirrer at 500 rpm and 25 °C for 1 h to ensure full hydration, which was characterized by the absence of visible solids.

### 2.3. Emulsion Preparation

**Figure 2:**
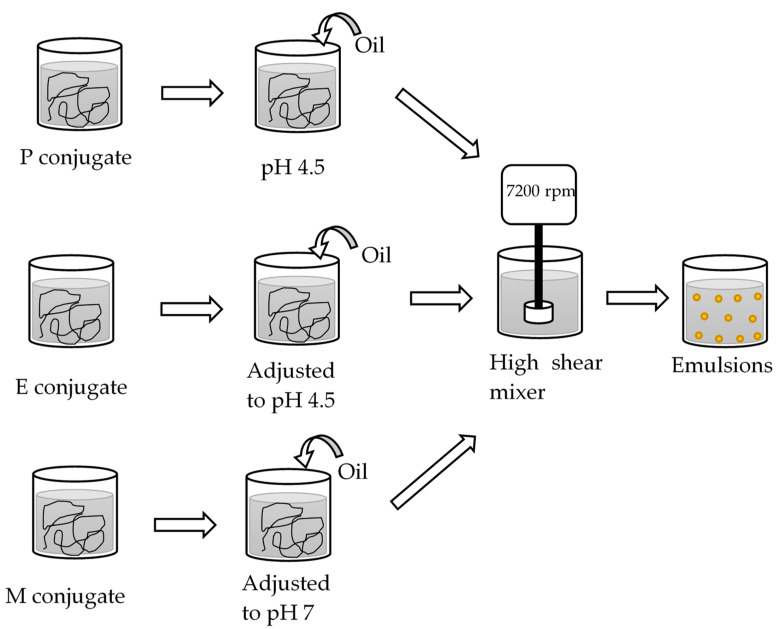
Flow diagram of the preparation of the P, E, and M emulsions.

Before the oil-in-water emulsions were prepared as summarized in (Figure 2), the oil phase was pre-treated to remove naturally present surface-active molecules to ensure all data could be interpreted as the result of the interfacial properties of the SBP–SC emulsifier systems. First, 500 mL of sunflower oil was mixed with 20 g of Florisil powder and stirred for 30 min, followed by centrifugation for 30 min at 2880 *g* (CR3i multifunction centrifuge, Waltham, MA, USA), both at 25 °C. The supernatant was recovered and stored in a dark glass bottle at 20 °C. Before use, it was then checked that the interfacial tension at the oil–water interface was constant (30.0 ± 0.4 mN.m^−1^ at 20 °C). The interfacial tension measurement method is introduced below.

The emulsions were then prepared as follows: 90 g of the P, E, or M emulsifier dispersion, and 10 g of the pre-treated oil were emulsified via high shear overhead mixing (Silverson L4M fitted with emulsor screen, Chesham, UK at 7000 rpm for 5 min at 20 °C. These emulsions were labelled emulsion P, E, and M. The processed emulsions were adjusted to pH 4.5 or pH 7 with 1 M HCl or 1 M NaOH, as appropriate, following preparation at the pH of the emulsifier system. Three independent replicate emulsions were prepared and each stored in separate glass vials at 25 °C until analysis on days 1 and 14 after preparation.

### 2.4. Analytical Methods

#### 2.4.1. Emulsion Droplet Size

A laser diffraction particle size analyzer (LS 13 320, High Wycombe, UK) was used to acquire emulsion droplet size distributions. The emulsion samples were gently shaken in their storage vials and a few droplets, as indicated by the instrument’s software, were added into the water contained in the dispersion cell of the equipment. Duplicate scattering patterns were acquired at 20 °C. These were automatically averaged and analyzed by the software, having set the refractive index for the sunflower oil and water to 1.465 and 1.333, respectively. The results are presented as volume-based density distribution and mean droplet size (d_4,3_).

#### 2.4.2. Zeta (ζ)-Potential

To determine the ζ-potential, the emulsions were gently shaken in their storage vials, followed by diluting to 0.04% *w/w* with water. After this, a particle electrophoresis instrument (Delsa Nano C, High Wycombe, UK) was used to acquire the ζ-potential data at 20 °C.

#### 2.4.3. Interfacial Tension 

The interfacial tension of 0.4% *w/w* SC solution and the three emulsifier systems at the oil/water interface was measured with the pendant drop method, using a profile analysis tensiometer (PAT-1, Sinterface Technologies, Berlin, Germany) at 20 °C. A drop of the water phase, as used for emulsification and at pH 7, with the cross-sectional surface area set to remain constant at 25 mm^2^, was suspended via a stainless steel capillary (4 mm diameter) into the oil phase, contained in a glass cuvette. Then, the interfacial tension was recorded.

#### 2.4.4. Viscoelastic Properties of the Emulsion Cream Phases

As noted in the introduction, bulk rheological data on the emulsion cream phases were acquired. To separate the cream phases, the emulsions were stored in a cylindrical separation funnel at 25 °C for 24 h, followed by draining off the serum phases. The viscoelastic properties of the cream phases were then analyzed using a shear rheometer (MCR 301, Anton Paar, Graz, Austria) fitted with a cone and plate geometry (ø 50 mm, 2° cone angle). Strain-controlled oscillatory amplitude sweeps (0.01–100%) at a constant frequency of 1 Hz were performed at 20 °C, and 25 logarithmically spaced data points were recorded. The storage modulus (G′) and loss modulus (G″) are reported as a function of amplitude.

### 2.5. Statistical Analysis

All data are reported as means ± standard deviations of triplicate freshly prepared samples. The data were statistically analyzed for significant difference (*p* < 0.05) by applying Analysis of Variance (ANOVA) using SPSS (IBM, Endicott, NY, USA).

## 3. Results

The emulsifying properties of the three different SBP–SC (1:1) emulsifier systems, namely the electrostatically stabilized (P), enzyme-catalyzed (E), and Maillard reaction (M) complexes, were examined. Oil-in-water (o/w) emulsions with 10% *w/w* oil and 0.4% *w/w* emulsifier in the aqueous phase were prepared and their physico-chemical properties and stability over 14 days were tested. Due to the pH of the different aqueous phases, the “natural” pH of the emulsion P and E was pH 4.5 and pH 5, respectively. The pH of the aqueous phase with the M conjugates was adjusted to pH 7 before emulsion preparation, as the M complexes were highly aggregated at acidic pH and no stable emulsion could be prepared. Following preparation, the pH of each emulsion was adjusted to pH 4.5 and pH 7, as acidic and neutral product environments are both relevant to application in foods. 

### 3.1. Physico-Chemical Emulsion Properties

#### 3.1.1. Size Distributions

**Figure 3:**
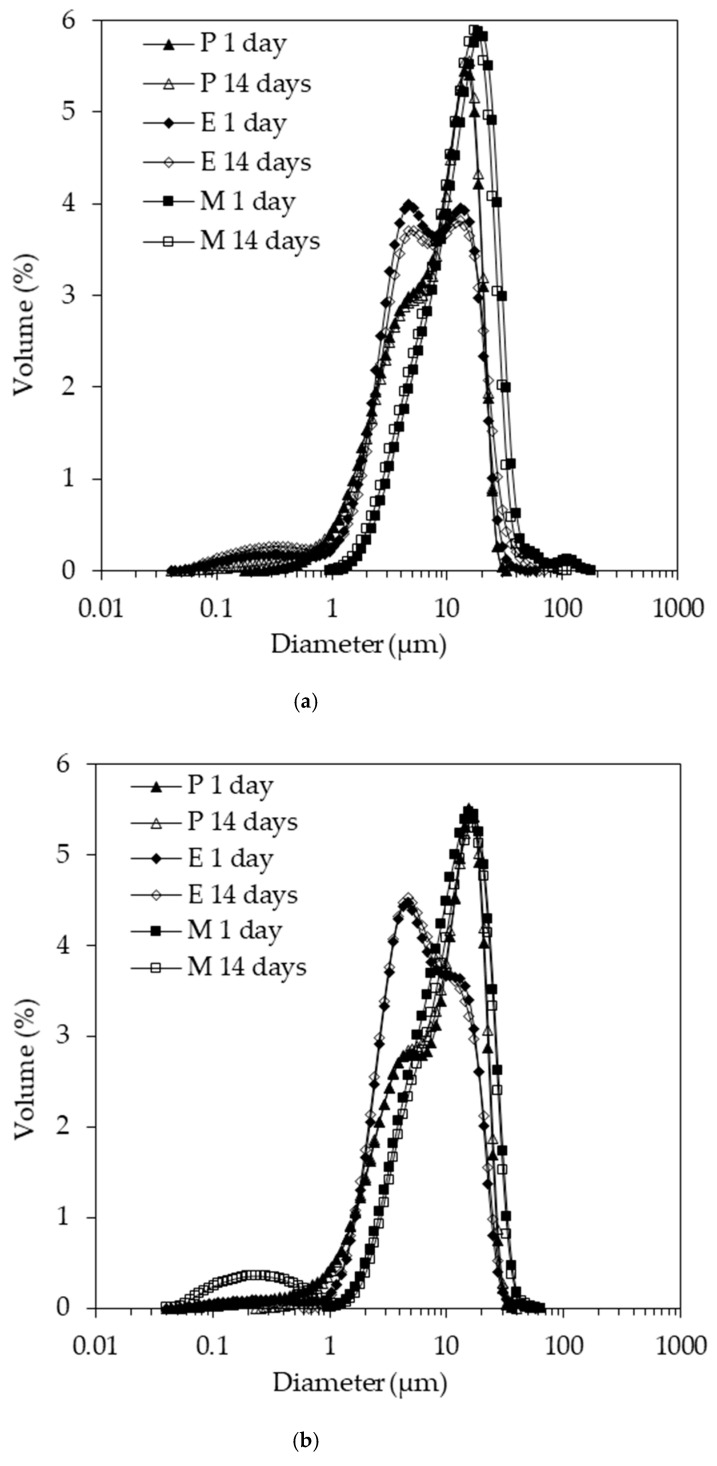
Droplet size distribution for the P, E, and M emulsions at (**a**) pH 4.5 and (**b**) pH 7 acquired at 20 °C 1 and 14 days after emulsion preparation, respectively. The values are the means of triplicate measurements of the freshly prepared samples. The standard deviation was within the size of the data points and is not shown.

**Figure 4:**
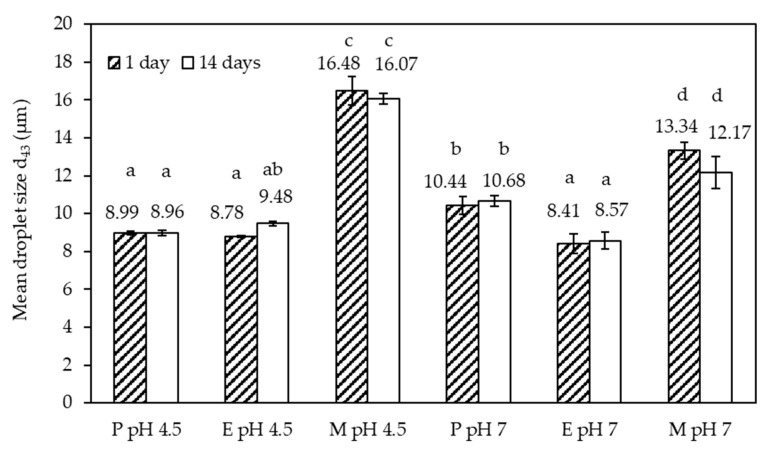
Mean droplet size data of the size distributions shown in (Figure 1). Different letters indicate significant differences between the data (*p* < 0.05).

The emulsion droplet size distributions acquired 1 and 14 days after emulsion preparation are depicted in (Figure 3). The data in (Figure 3a) relate to pH 4.5, while the data in (Figure 3b) relate to pH 7. The volume-based mean size data of these distributions are summarized in (Figure 4). The shapes of the droplet size distributions were dissimilar. Leaving aside the minor fractions below 1 µm and above 30 µm, the size distribution of emulsion P featured a shoulder before peaking, while the distribution of emulsion E was characterized by two peaks, and emulsion M had a monomodal distribution, with the whole size distribution shifted slightly to larger droplet sizes. This shift may have been due to the slightly lower interfacial activity of emulsifier M compared to emulsifiers P and E (see below). The height of the two peaks for emulsion E was similar at pH 4.5, whereas the smaller population had a higher peak at pH 7. The first peak was located around the same size range as the shoulder for emulsion P. It appears that the P and E emulsifiers were more effective in stabilizing the process-induced droplet sizes. The reason for the creation of the two main populations in emulsions E and P, as indicated by the two peaks and the shoulder, respectively, would need to be investigated in further emulsification trials to understand whether it is process- or formulation-induced. Although we reported in our previous study that the z-average for emulsifier P at pH 4.5 was significantly larger than for emulsifier E [25], the behavior at the oil–water interface was largely comparable as discussed further below. Although this comparability makes the following hypothesis unlikely to be correct, it is worth bringing forward since the interfacial tension analysis reported here could be complemented by a more comprehensive set of data, including a range of emulsifier concentrations and interfacial rheology data, as well as compositional analysis. Therefore, in the case of emulsion E, the more pronounced two-peak shape and the larger fraction of the smaller droplet size population could have also been due to a range of interfacial activities present in this emulsifier system. This would be the result of the fact that enzymatic crosslinking during emulsifier preparation was unlikely to be limited to creating SC–SB conjugates, as reported in our previous study [25]. Laccase can also catalyze crosslinks between SBP molecules [26], and SC molecules [27]. 

The size distributions acquired 1 and 14 days after the emulsion preparation overlapped in good approximation, except for emulsion M at pH 7. A clearly discernible small size population was recorded. This population is the reason the mean size data plotted in (Figure 4) show a small but non-significant decrease (*p* < 0.05) in d_4,3_ for emulsion M at pH 7. Due to the complex shape of the size distributions, the mean value should not be overinterpreted. It is worth noting though that emulsions P and E had similar d_4,3_ values at both pH values, approximately 8–10 µm. The value was higher for both M emulsions, with approximately 16 µm at pH 4.5 and approximately 12 µm at pH 7. This observation alone suggests that the Maillard complexes of SBP–SC were less effective at emulsifying oil than the electrostatically conjugated (P) and enzymatically crosslinked (E) SBP and SC. 

It should be noted that the size distributions were based on light scattering data with droplets as well as emulsifier aggregates contributing to the measurement signal. We reported previously the aggregate size at pH 4.5 for P conjugates and E complexes as being approximately 200 nm, while it was approximately 2 µm for M complexes [25]. The distributions for the M emulsion at pH 7 showed a minor broad population below 1 µm, likely the result of non-adsorbed emulsifier aggregates. We previously reported that at pH 7 all three emulsifier systems had a ζ-average of approximately 200 nm [25]. Therefore, the minor broad population between 0.1 and 1 µm, noted for emulsion M 14 days after preparing this emulsion at pH 7, can be attributed to non-adsorbed emulsifier. This population was absent one day after emulsion preparation, which may be due to the initially loose association of the non-adsorbed emulsifier with the droplets. Indeed, the main population was shifted to slightly larger diameters on day 1 compared to day 14. Micrographs presented in the supplementary Material (see Figures S1 and S2) demonstrate that the microstructure of this particular emulsion, M prepared at pH 7, appeared flocculated on day 1, but not on day 14. Finally, emulsion M at pH 4.5 showed a minor fraction with a peak of around 100 µm. We attribute this observation to coalescence due to the aforementioned instability of the Maillard complexes at pH 4.5.

#### 3.1.2. ζ-Potential

**Figure 5:**
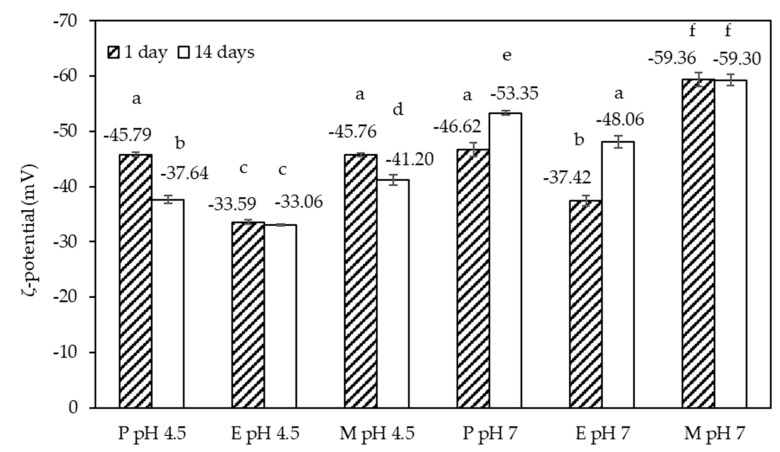
The influence of pH conditions on the zeta (ζ)-potential for the P, E, and M emulsions at pH 4.5 and pH 7 after 1 (filled bars) and 14 (open bars) days at 20 °C. The values are means, and the error bars correspond to a ± 1 standard deviation of triplicate measurements of freshly prepared samples. Different letters indicate significant differences between the data (*p* < 0.05).

The ζ-potential values acquired on the six emulsions 1 and 14 days after preparation are shown in Figure 5. The values were negative with an absolute value of at least 30 mV, which is the absolute value above which emulsions are typically stable [28]. Emulsion E at pH 4.5 had the lowest net charge of just over 30 mV. Similar to emulsion M at pH 7, showing the highest net charge of all emulsion samples, the ζ-potential value remained constant over the storage period. The other four emulsions, on the contrary, showed a significant change in the ζ-potential value, which is indicative of significant molecular re-arrangements at the emulsion interface, or within the non-adsorbed fraction of the emulsifier system. Across the whole data set, the absolute values of the ζ-potential appeared slightly higher at pH 7 compared to pH 4.5, congruent with our previous observation for the emulsifier systems [25]. It should be pointed out that the emulsions were processed at the natural pH value of the emulsifier systems, followed by the adjustment of the pH to pH 7. The natural pH values of the emulsifier systems were pH 4.5 and pH 7, respectively. An increase in the absolute values of the ζ-potential, as the result of adjusting the pH from an acidic pH to pH 7, has previously been reported for a β-lactoglobulin–SBP [21], and a fish skin gelatin–SBP [29] stabilized emulsion. In the following, an attempt is made to explain the changes in the ζ-potential noted over time. 

In the case of emulsion P, prepared at pH 4.5, the magnitude of the ζ-potential decreased over time, but this has not affected the droplet size distribution (see Figure 4), or emulsion microstructure (see Figure S1). It appears that this observation was linked to our previous finding that this emulsifier system slightly aggregated over time [25]. The decrease in the absolute value of the ζ-potential for emulsion M, prepared at pH 4.5, was probably due to the instability of the Maillard complexes at this pH [25], which also led to partial coalescence of the emulsion M, as discussed above. The observed changes in the ζ-potential at pH 7 were of the opposite sign, i.e., the actual ζ-potential values decreased, and the absolute value increased between day 1 and day 14. The two emulsions concerned were emulsions P and E. This change may be explained by the fact that these two emulsions were prepared at the acidic pH of their respective emulsifier system and slow interfacial re-arrangements due to changing the conditions for SC from uncharged to charged. 

### 3.2. Interfacial Tension at pH 7

**Figure 6:**
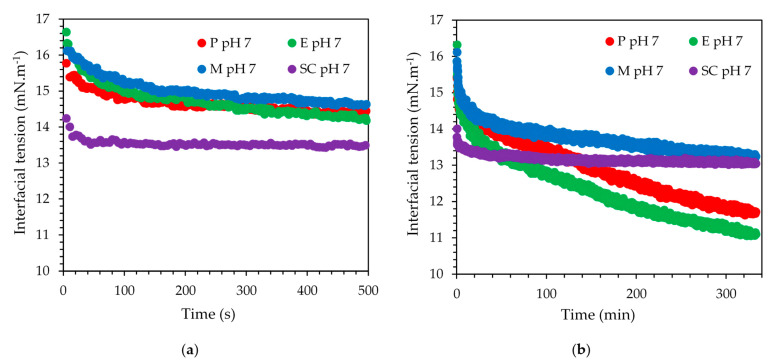
Interfacial tension for the 0.4% *w/w* SC (purple), P (red), E (green), and M (blue) dispersions at pH 7 at the oil–water interface at 20 °C. (**a**) Data for the first 600 s and (**b**) the complete data set with time in minutes.

The interfacial tension data for SC and the three mixed emulsifier systems are shown in (Figure 6) on two time scales to highlight the short- and the long-term behavior. All data correspond to pH 7 solution conditions only, as the Maillard reaction-based emulsifier system showed rapid sedimentation at pH 4.5, thereby hampering the acquisition of interfacial tension data with the selected method. The first data point of each curve presented in Figure 6 was recorded 5 s or later after the creation of the interface, explaining why the starting value was not 30 mN·m^−1^, which was the interfacial tension value of the bare interface. A steep initial decrease of the interfacial tension was shown by SC and a constant value was reached within less than 100 s. P and E showed comparable short-term kinetics, and their data traces overlapped. However, instead of levelling off, the interfacial tension continued to decrease, eventually dipping in value below the equilibrium value recorded for SC alone. Close to the end of the chosen measurement duration, their interfacial tension values levelled off at 11.5 mN·m^−1^ compared to 14 mN·m^−1^ for SC. The Maillard reaction-based emulsifier showed the slowest kinetics, asymptotically reaching the interfacial tension value of SC over the duration of the measurement. This slow kinetics implies a larger molecular weight compared to the other three emulsifiers. More than one SBP may crosslink with SC during the Maillard reaction [30]. Hence, the molecular weight may be higher compared to the electrostatically stabilized or enzymatically crosslinked emulsifier systems where SC and SBP combine as 1:1 [31,32]. Clearly, the short-term interfacial tension behavior was dominated by the SC component of any of the three systems, followed by adsorption of the SBP component to further slowly decrease the interfacial tension over a long period of time.

### 3.3. Oscillatory Rheology of the SBP–SC Emulsion Cream Phases

**Figure 7:**
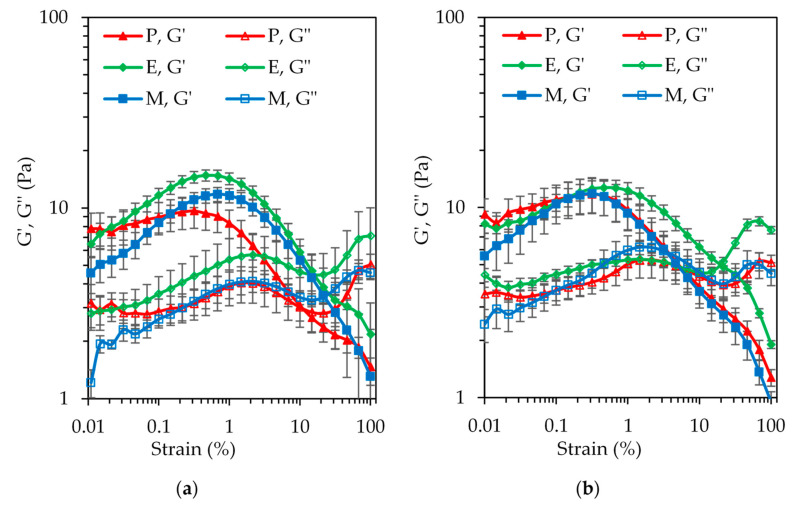
The storage modulus (G′l filled symbols) and loss modulus (G″; open symbols) at (**a**) pH 4.5 and (**b**) pH 7 viscoelastic moduli at a constant frequency of 1 Hz for the emulsion cream phase of P (triangle), E (diamond), and M (square) emulsions one day after preparation at 20 °C. The values are means, and the error bars correspond to a ± 1 standard error in freshly prepared samples.

Figure 7 shows the results of the amplitude sweep tests on the emulsion cream phases at pH 4.5 and pH 7. In the range of the strain amplitudes probed, only the cream phase of emulsion P at pH 4.5 showed a plateau value for the storage modules G′ at the very low end of this range. At pH 7, the first data point was higher than the second data point, similarly for the cream phase of emulsion E at this pH, which is most likely a measurement artefact. None of the cream phases at pH 7 exhibited a linear viscoelastic domain in the strain amplitude range applied. The G′ values steadily increased (following the plateau in case of cream phase P at pH 4.5), undergoing a maximum at a strain between 0.3% and 0.5% before rapidly declining. There was evidence of a measurement artefact, most likely slip, at high strain amplitudes for all the samples. These high strain amplitudes outside the small deformation domain were applied to detect the crossover with the loss modulus G″, which was indeed observed for all six samples. The shape of G″ was similar to the shape of G′, but peaking at a higher strain amplitude and being overall lower until the crossover.

An overshoot of G′ and G″ at an intermediate strain amplitude is known as type IV behavior in large amplitude oscillatory shear (LAOS) rheology [33]. It should be noted that concentrated emulsions more typically show type III behavior, characterized by a G′ plateau and a G″ overshoot. It appears that the cream phases of this study not only showed strain hardening, but additional interactions may exist between the hydrophobic groups of the emulsifier systems. The kinetics of the resulting microstructure evolution seemed to be faster than the deformation rate [34], as well as more pronounced for cream phases E and M at pH 4.5 compared to P. This can be explained by the fact that in the case of the P emulsifier, the hydrophobic groups were buried or adsorbed at the o/w interface. Hence, interaction upon bringing the droplets close together through creaming were limited. During the enzymatic (laccase-catalyzed) or chemical (Maillard reaction) reaction, on the contrary, to create the E and M emulsifier systems, the structures of both the SBP and SC biopolymers were rearranged to promote crosslinking. This led to the exposure of an abundance of hydrophobic groups on the emulsifier surface, not all of which were involved in the stabilization of the emulsion interface. Upon bringing the droplets close together through creaming of the initially prepared dilute emulsion, these available hydrophobic groups were able to interact, leading to the observed response in the oscillatory shear rheology test. 

At pH 4.5, the cream phase of emulsion E showed the highest G′ values in the lead up to the overshoot. This observation may have been the consequence of a higher oil volume fraction in this cream phase compared to P and M, which is entirely possible due to the significantly lower absolute ζ-potential value of this emulsion (Figure 5). The value was also close to the 30 mV threshold, suggesting the possibility of aggregation in the cream phase, although the presence of aggregation could not be clearly deduced from the micrographs in Figure S1. In terms of the G′ and G″ crossover strain, cream phase P showed an earlier transition from elastic- to viscous-dominated behavior. This observation is congruent with the lesser pronounced G′ overshoot, suggesting a weaker overall microstructure. At pH 7, the data for the three cream phases practically overlapped, in accordance with the interfacial rheology data.

## 4. Conclusions

Three SBP and sodium caseinate-based mixed emulsifiers systems were assessed in their ability to stabilize o/w emulsions. The emulsifier systems were prepared through electrostatic attraction, enzymatic crosslinking, and Maillard reaction at the biopolymer ratio of 1:1 SBP:SC. All three emulsifier systems successfully stabilized the emulsion interfaces against coalescence leading to bulk phase separation at pH 4.5 and pH 7 for at least 14 days of storage. In fact, only the emulsion prepared with the Maillard reaction-based emulsifier system applied at pH 4.5 was unstable to partial coalescence. All other emulsions were successfully stabilized against coalescence. All emulsions had ζ-potential values indicative of long-term stability, except for the emulsion prepared with the enzymatically crosslinked emulsifier at pH 4.5. Although the absolute value for this emulsion was close to the generally accepted stability threshold of 30 mV, no significant difference (*p* < 0.05) in the mean droplet size was observed during the assessed storage period of 14 days. Regarding interfacial tension data acquired at pH 7, the Maillard-based emulsifier was less effective compared to the other two emulsifiers. The precipitation of emulsifier M at pH 4.5 meant that a similar set of data was not acquired at pH 4.5. However, the small deformation oscillatory shear behavior of the three emulsion cream phases that were recovered from the pH 4.5 emulsions differed in contrast to the pH 7 cream phases. Elastic behavior dominated, and the data suggest a contribution by the emulsifier system. The emulsifier system based on enzymatic crosslinking imparted the strongest microstructure, while the weakest microstructure was found for the emulsion stabilized with the emulsifier system based on electrostatic attraction. 

In conclusion, the emulsions prepared with either of the SBP–SC mixed emulsifier systems studied here at two pH values relevant to food emulsions—acidic and neutral—were largely stable against coalescence for at least 14 days, independent of the interaction mechanism between the two biopolymers. However, the emulsion based on the enzymatically crosslinked conjugate was the most acid-tolerant compared to the other two emulsions, stabilized by electrostatically interacted SBP–SC and Maillard reaction product conjugates, respectively. In food applications, the electrostatically interacted conjugates might be the safest. Laccase is a fungal enzyme that might cause concern regarding allergenicity [35], and the Maillard reaction might produce advanced glycation end-products (AGEs), causing side-effects in healthy individuals [36,37]. Nevertheless, out of the three SBP–SC-based emulsifier systems studied, the enzymatically crosslinked conjugates might be most beneficial for application in acidic food products characterized by a firm texture. 

## Data Availability

Not applicable.

## Supplementary Materials

The following are available online at https://www.mdpi.com/2304-8158/10/3/631/s1, Figure S1 Optical microscopy visualized microstructure for P emulsion (a) one day and (d) 14 days; E emulsion after (b) one day and (e) 14 days; M emulsion after (c) one day and (f) 14 days at pH 4.5 at 20 °C. The scale bar in all micrographs corresponds to 400 µm., Figure S2. Optical microscopy visualized microstructure for P emulsion (a) one day and (d) 14 days; E emulsion after (b) one day and (e) 14 days; M emulsion after (c) one day and (f) 14 days at pH 7 at 20 °C. The scale bar in all micrographs corresponds to 400 µm.
