# Supplementary material for: Impact of Type of Sugar Beet Pectin–Sodium Caseinate Interaction on Emulsion Properties at pH 4.5 and pH 7
Source: Foods. 2021 Mar 17;10(3):631. doi: 10.3390/foods10030631 (PMC8002390; doi:10.3390/foods10030631)
Supplement: Supplementary file 1 [file foods-10-00631-s001.pdf]

foods

Supplementary material

Optical microscopy of emulsions

The microstructure of the emulsions was assessed via optical microscopy. For sampling, the storage glass vial was gently inverted a few times to carefully break up any cream layer. A small quantity of emulsion sample was then placed on a glass slide and covered with a glass cover slide before capturing images with the digital camera set up of an optical microscope (EVOS f1, Thermo Fisher Scientific, Loughborough, UK), operated in brightfield illumination mode. Temperature of assessment was 25 °C.

Figure S1 shows the microstructure of the emulsions at pH 4.5 as it appeared one day and 14 days after preparation. The micrographs reveal a slightly flocculated microstructure with no obvious change in microstructure over storage for any of the three emulsion. The preparatory conditions of P and E were pH 4.5 and pH 5, respectively. As there was a high level of aggregation of SBP–SC conjugates at pH 4.5, the M emulsion was prepared at pH 7. As depicted in the microstructure results in Figure S2, both P and E emulsions at pH 7 were stabilized, something which was also observed for emulsions at pH 4.5. These results suggest that the microstructure of both P and the E emulsions were the least affected when the pH was adjusted. In contrast, the M emulsion possessed a flocculated microstructure after preparation, which decreased over the period of 14 days indicating that there was a structural rearrangement within emulsions during storage at pH 7.

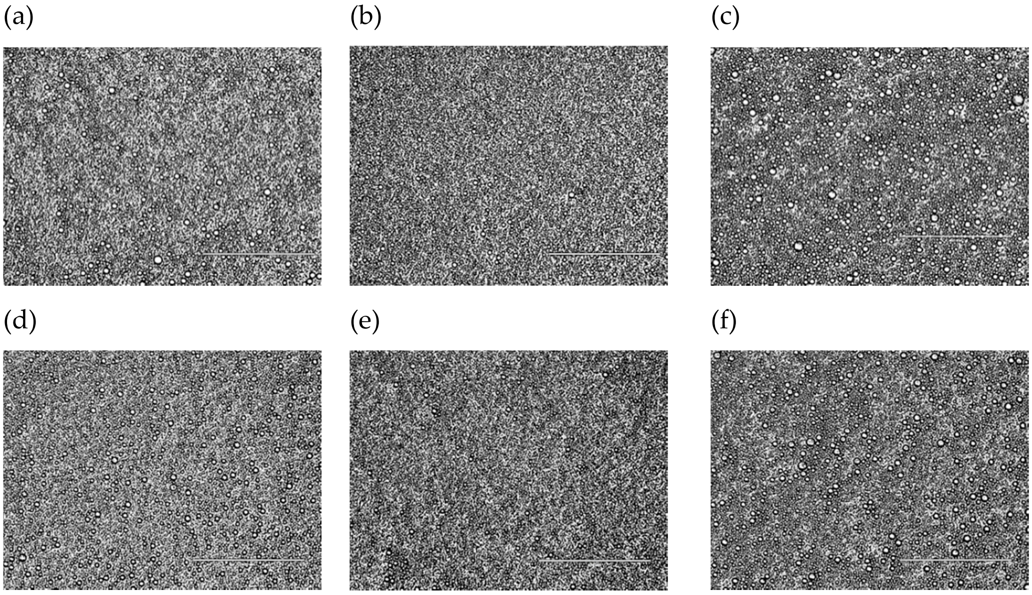

Figure S1. Optical microscopy visualized microstructure for P emulsion (**a**) one day and (**d**) 14 days; E emulsion after (**b**) one day and (**e**) 14 days; M emulsion after (**c**) one day and (**f**) 14 days at pH 4.5 at 20 °C. The scale bar in all micrographs corresponds to 400 μm.

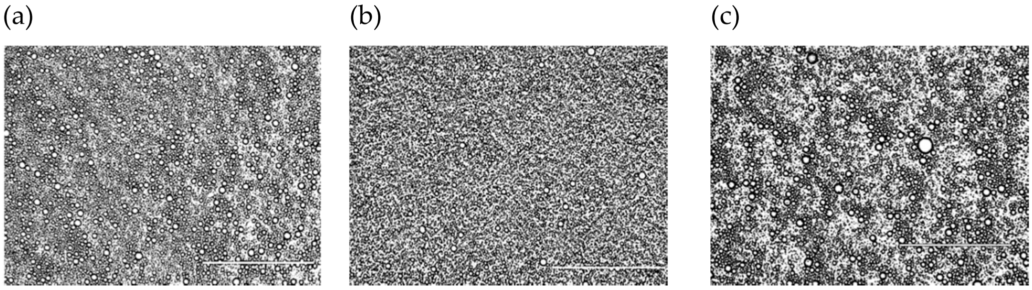

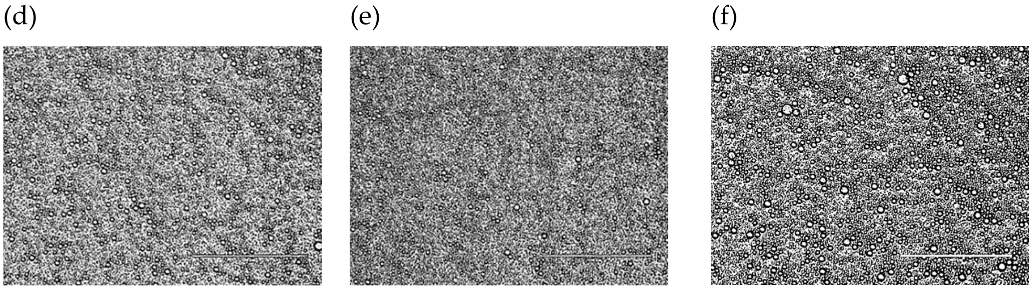

Figure S2. Optical microscopy visualized microstructure for P emulsion (**a**) one day and (**d**) 14 days; E emulsion after (**b**) one day and (**e**) 14 days; M emulsion after (**c**) one day and (**f**) 14 days at pH 7 at 20 °C. The scale bar in all micrographs corresponds to 400 μm.
